# Nasal Septal Ancient Schwannoma: Ancient and Rare

**DOI:** 10.7759/cureus.44294

**Published:** 2023-08-28

**Authors:** Rathakrishnan Venkatasamy, Avatar Singh Mohan Singh, Kanivannen Arasu, Salina Husain, Chew Mianxin

**Affiliations:** 1 Department of Otolaryngology - Head and Neck Surgery, National University of Malaysia, Kuala Lumpur, MYS; 2 Department of Otorhinolaryngology - Head and Neck Surgery, Taiping Hospital, Taiping, MYS; 3 Department of Otolaryngology - Head and Neck Surgery, Taiping Hospital, Taiping, MYS; 4 Department of Otolaryngology - Head and Neck Surgery, University Kebangsaan Malaysia Medical Center, Kuala Lumpur, MYS; 5 Department of Pathology, Taiping Hospital, Taiping, MYS

**Keywords:** transnasal endoscopic surgery, septal neoplasm, schwann cell neoplasm, ancient schwannoma, septal schwannoma

## Abstract

Schwannoma is a rare benign neurogenic tumor arising from the Schwann cells of peripheral nerves. A 77-year-old man presented with progressively worsening left nasal block and hyposmia for the past six months. Nasal endoscopy revealed a polypoidal reddish mass occupying the left middle meatus. The biopsy was in favor of ancient schwannoma. Endoscopic transnasal excision of the mass arising from the left nasal septum was performed. A middle meatal antrostomy was also performed. The tumor cells were positive for S100 protein. Presenting symptoms are common to other sinonasal tumors, and the differential diagnoses include carcinoma, inverted papilloma, sarcoma, lymphoma, and neurofibroma. Schwannomas are composed of spindle cells with two histologically distinct patterns that can be mixed: Antoni type A and Antoni type B. A neural crest marker antigen, S-100 protein, is useful to corroborate our diagnosis. It is vital to consider nasal septal schwannoma in the differential diagnosis of patients complaining of unilateral nasal obstruction with polypoidal nasal mass, especially the posterior third of the nasal septum. Transnasal endoscopic surgery is the preferred approach for nasal septal schwannoma.

## Introduction

Schwannoma, as the name suggests, arises from the Schwann cells of peripheral nerves. It is a benign and slow-growing neurogenic tumor [1,2]. The tumor has a high affinity for the head and neck region, and the incidence is reported to be between 25% and 45%. However, less than 4% of the cases occurred in the sinonasal tract [3]. Based on our search in the PubMed search engine, no report of nasal septal schwannoma in Malaysia was found. Further search for international cases published in the English language yielded a handful of cases since 1943. Two groups of authors have reviewed the cases of nasal septal schwannoma reported in English language literature. First, Berlucchi et al. published a case report with a review of the literature in 2000. A total of 12 cases including Berlucchi et al.’s case were reported [1]. Subsequently, Min et al. reviewed 19 cases published in the English language from the year 2000 till 2017 [2]. These cases were analyzed based on the presenting symptoms, endoscopic and radiological findings, and treatment modalities. Recently, we encountered a patient with nasal septal schwannoma, which was completely excised by endoscopic excision.

## Case presentation

**Figure 1:**
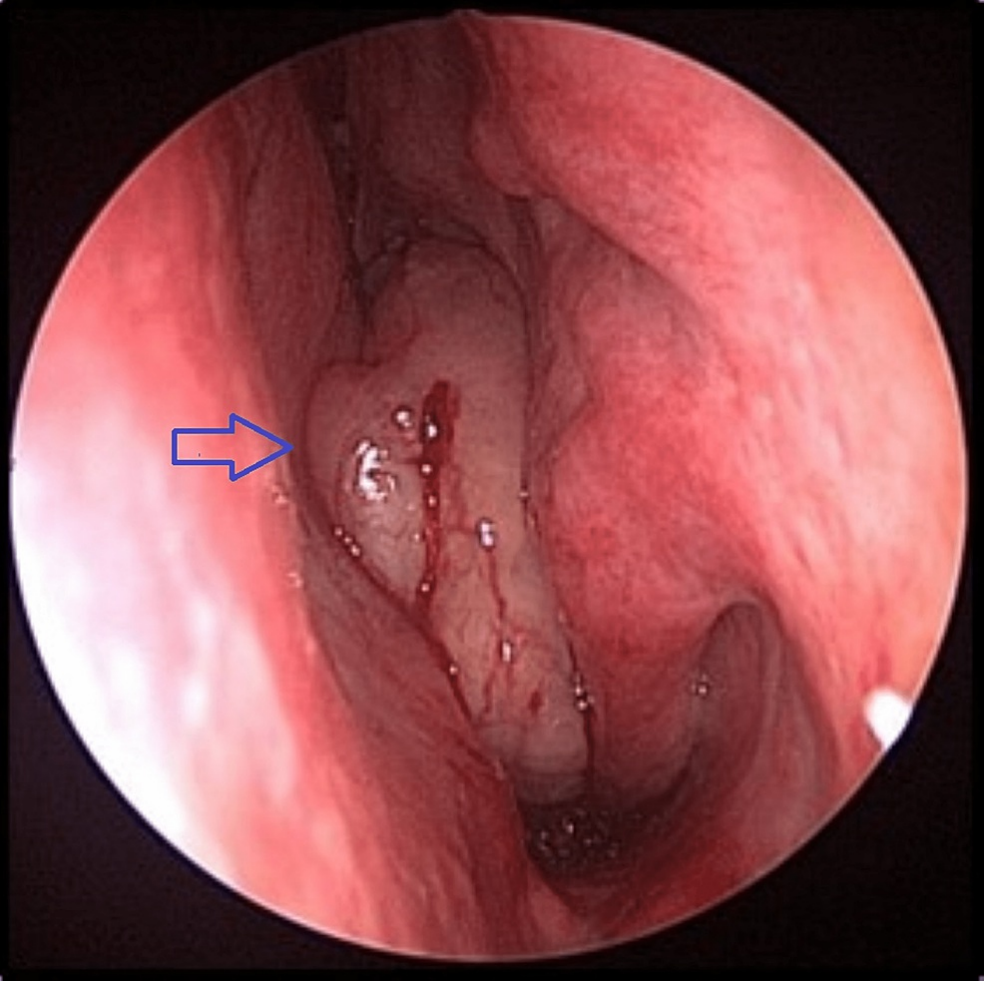
Nasal tumor (blue arrow) occupying the left nasal cavity between septum medially and inferior turbinate laterally.

A 77-year-old man presented with progressively worsening left nasal block for the past six months. It was associated with hyposmia. He was primarily concerned with persistent nasal block. The patient had to resort to mouth breathing occasionally, and nasal block was interfering with his sleep. He had no history of epistaxis, facial pain, and allergic symptoms. He denied constitutional symptoms. Neurological examination was unremarkable. No abnormalities were found during neck examination. He had neither café-au-lait spots nor cutaneous neurofibromas. Nasal endoscopy revealed a polypoidal reddish mass occupying the left middle meatus (Figure 1). There was contact bleeding, and a biopsy was taken before achieving hemostasis.

**Figure 2:**
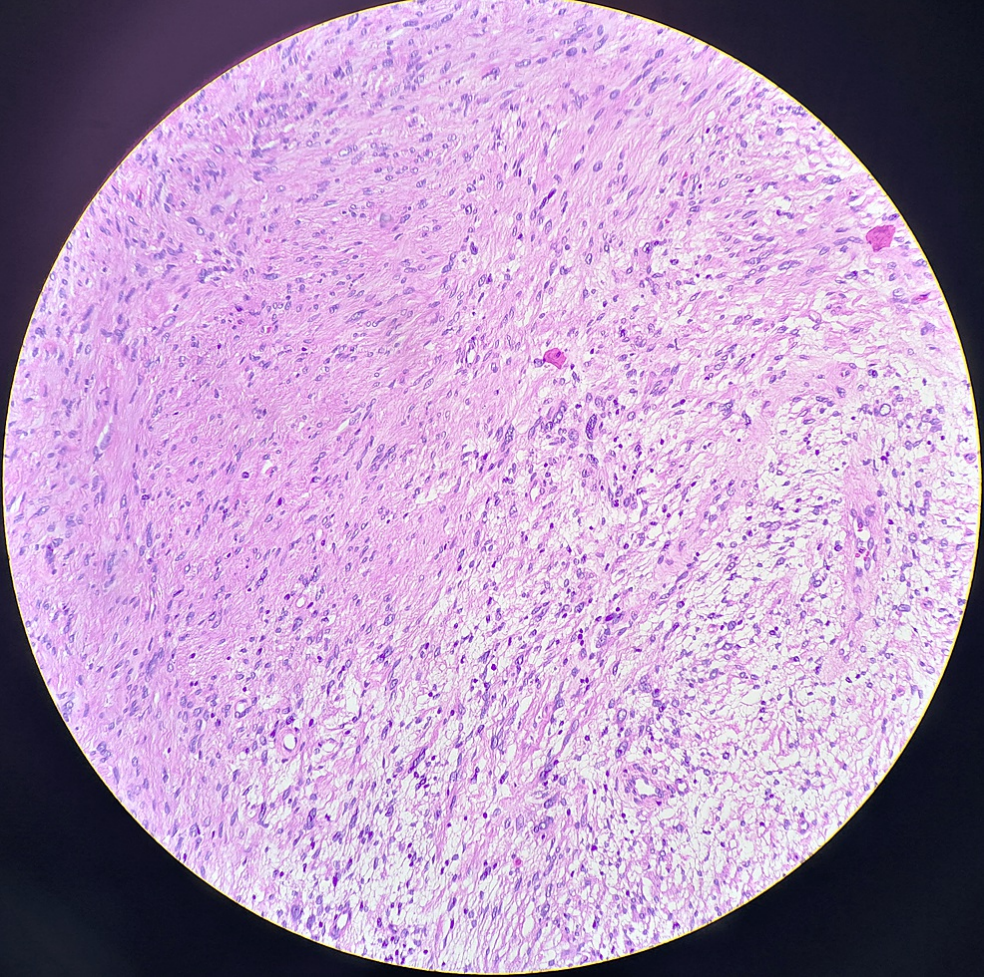
Alternating compact Antoni A (left) and loose Antoni B (right) areas.

**Figure 3:**
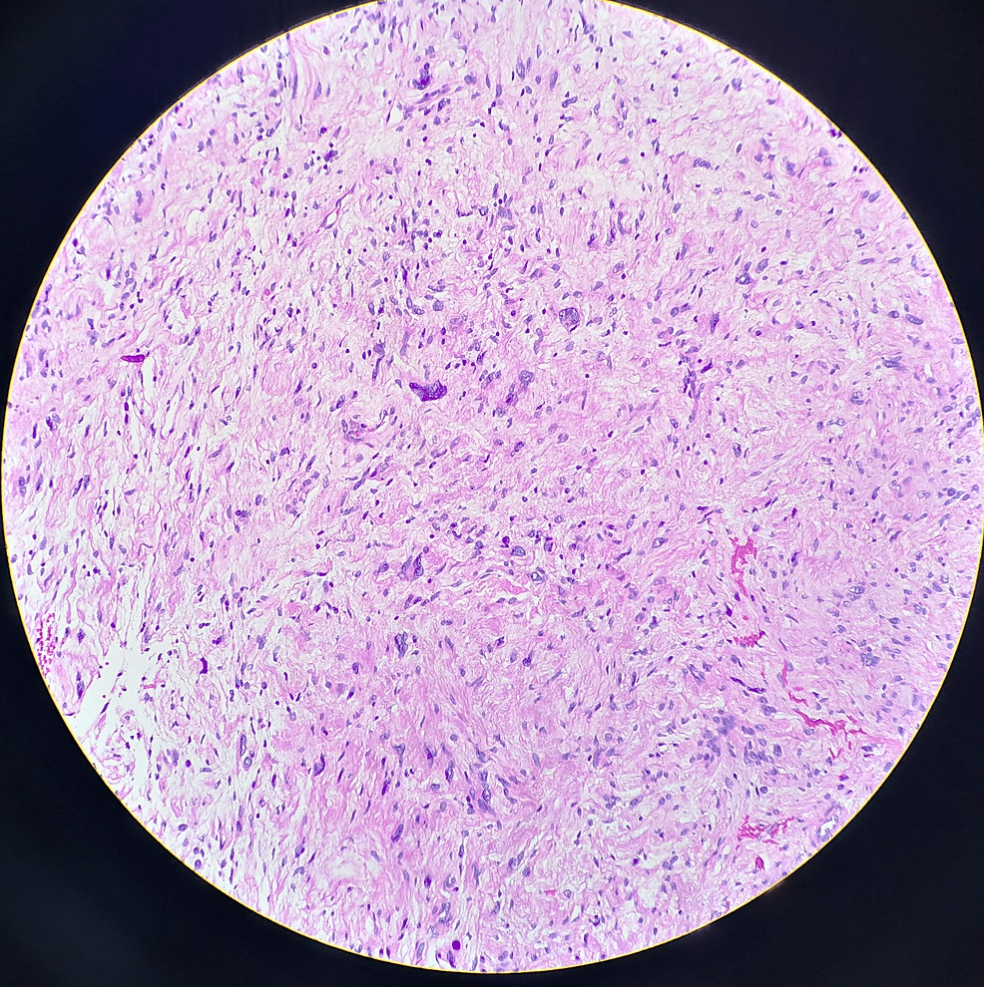
Ancient change characterized by the presence of scattered atypical nuclei of degenerative type.

Microscopic examination showed spindle-shaped cells arranged in interlacing fascicles. Nuclear palisading around the fibrillary process was occasionally seen. There were hypocellular areas composed of loosely arranged cells with round nuclei with indistinct processes (Figures 2, 3). The hypocellular tissues indicate degenerative changes. Hence, histopathological features were in favor of ancient schwannoma. No increase in mitosis, tumor necrosis, or evidence of malignancy was visualized. The tumor cells were positive for S100 protein and CD34.

**Figure 4:**
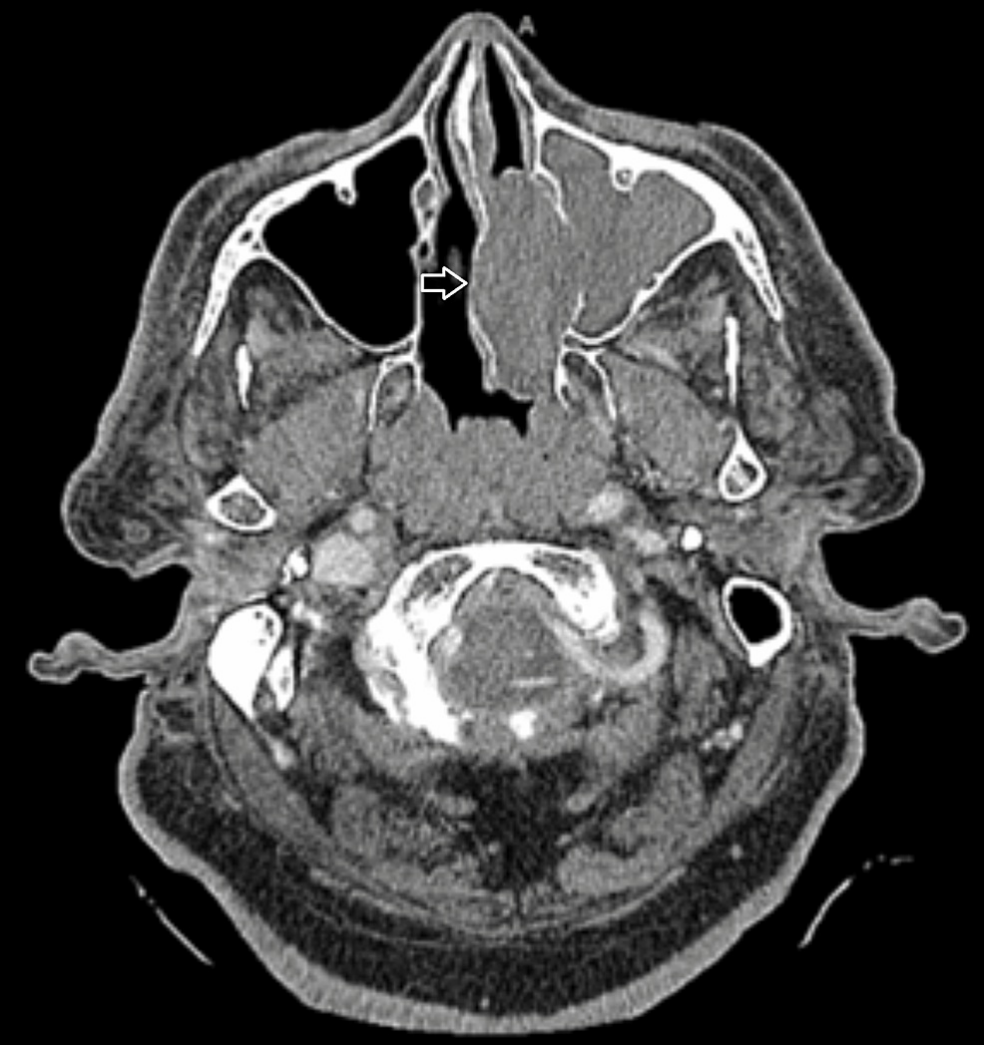
CT (axial view) of the paranasal sinuses showing non-enhancing soft tissue lesion in the left nasal cavity (arrow) extending to the left choana. The mass is causing mass effect and thinning of the nasal septum and medial wall of the left maxillary sinus. CT, computed tomography

**Figure 5:**
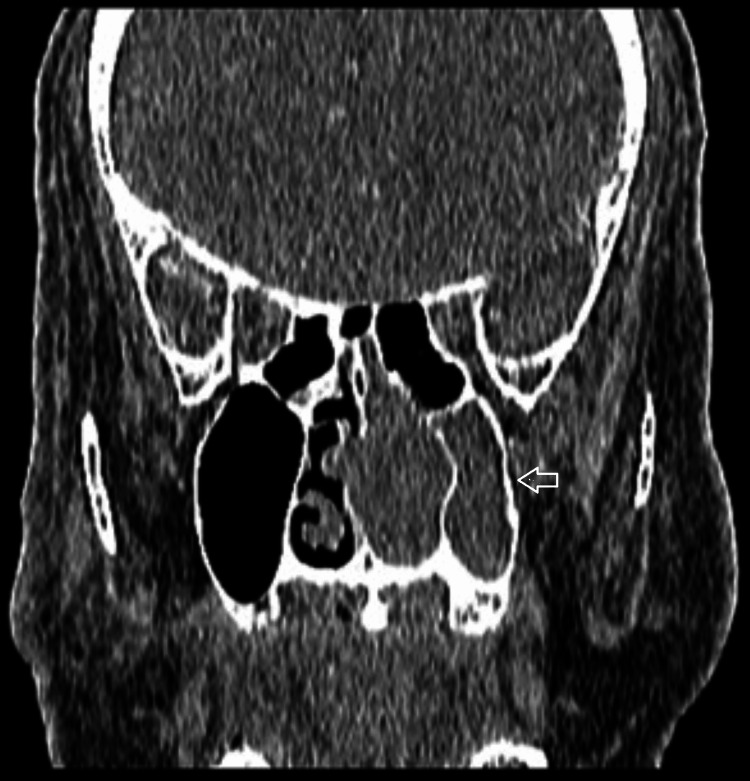
CT (coronal view) of the paranasal sinuses showing soft tissue lesion occupying the left nasal cavity with complete opacification of the left maxillary sinus (arrow). The left ostiomeatal complex is obstructed.

A computed tomography (CT) of the paranasal sinuses revealed a well-defined, non-enhancing, soft tissue density lesion occupying the left nasal cavity extending to the choana (Figures 4, 5). It was causing mass effect and thinning of the nasal septum and medial wall of the left maxillary sinus. No bony invasion was visualized.

Intra-operatively, a solid lobulated nasal mass with a pedicle arising from the middle part of the left nasal septum was identified. The mass was compressing the middle turbinate and extending to the choana. Endoscopic excision of the nasal mass was performed after securing the pedicle. A middle meatal antrostomy was performed. Maxillary and anterior ethmoid sinuses were free from the tumor.

**Figure 6:**
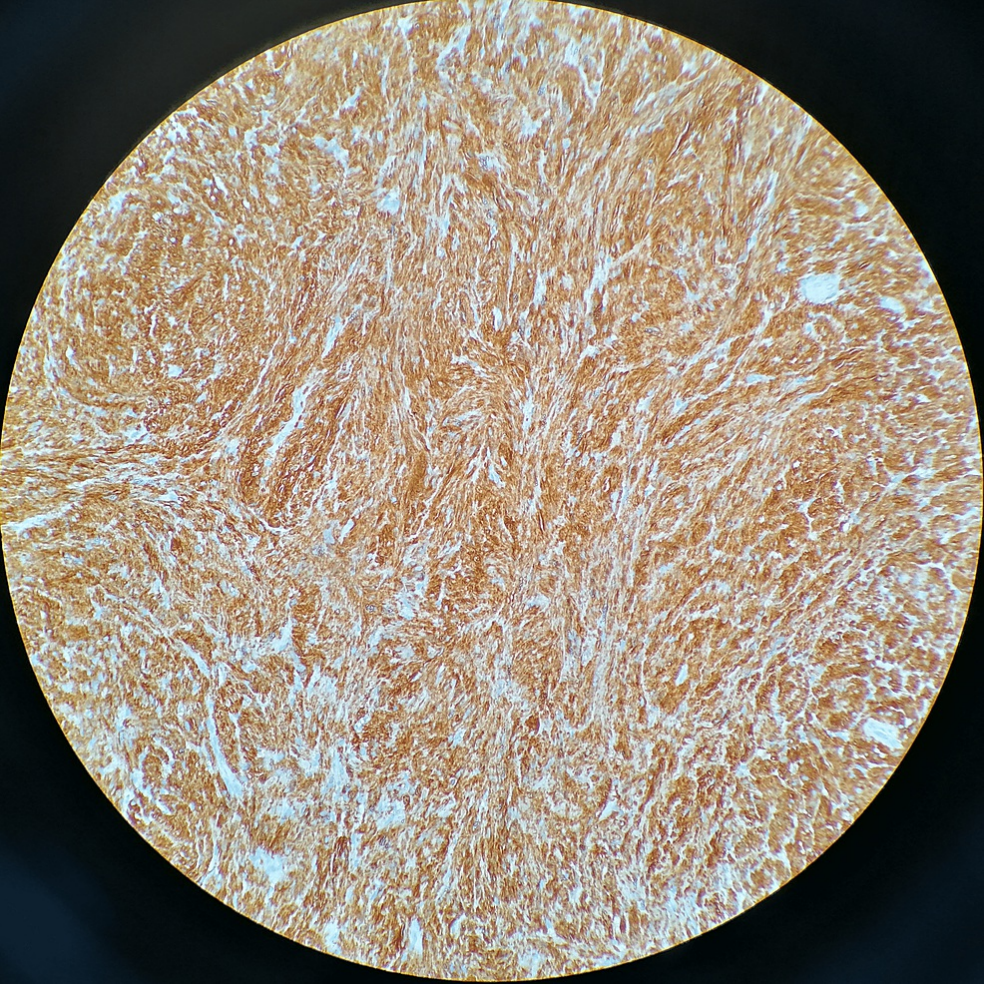
S100 protein expression is diffuse and strong in schwannoma.

**Figure 7:**
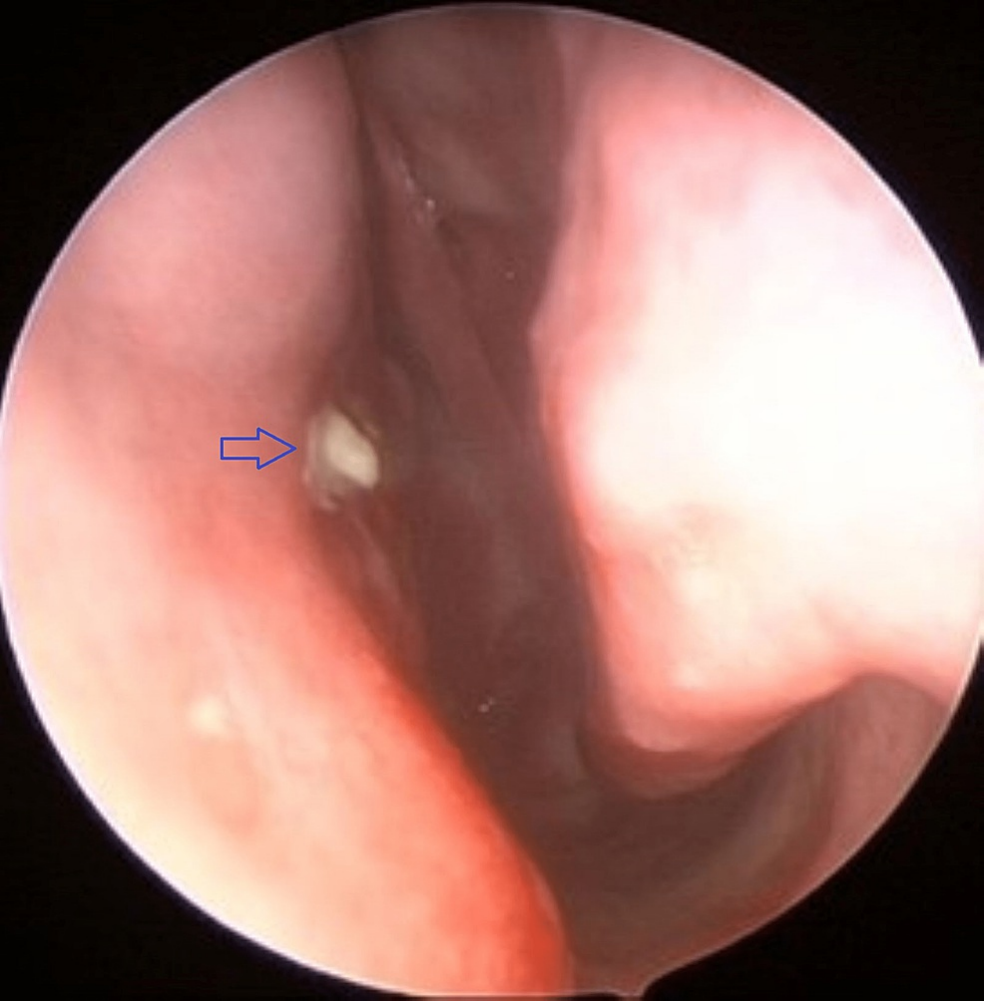
Nasal septum (blue arrow) a month after removal of the tumor.

The histopathology report of the nasal septal mass concurred with the previous biopsy report. The tumor cells were positive for S100 protein (Figure 6) and negative for smooth muscle actin and desmin. Thus, the diagnosis of ancient schwannoma was confirmed. The patient’s nasal mucosa was healing (Figure 7) and he is devoid of nasal blockage. The patient has been regularly seeing us for surveillance nasal endoscopy. Currently, it has been six months since his surgery, and no evidence of tumor recurrence is seen in nasal endoscopy.

## Discussion

Schwannoma is a benign tumor arising from the Schwann cells of the peripheral nerves. It has no predilection for age and sex. The age distribution ranges from three-month-old babies to 80s [1,2,4]. The symptoms may differ based on the site, and the presenting complaints of the patients are often nonspecific [5]. However, patients with nasal septal origin tend to become symptomatic at an earlier stage possibly due to the limited room for growth in the nasal cavity [6]. In our case, the patient presented with unilateral nasal block. Both Berlucchi et al.’s and Min and Kim’s data record nasal obstruction as the most common symptom followed by rhinorrhea and epistaxis. These are the symptoms common to other sinonasal tumors, and the differential diagnoses include carcinoma, inverted papilloma, sarcoma, lymphoma, and neurofibroma [4,5].

Based on previous reports, septal schwannoma usually manifests with polypoidal mass without any distinctive features [1,2,7]. Therefore, the differential diagnosis includes a wide spectrum of lesions such as angiomatous polyps to malignant tumors, melanoma, and olfactory neuroblastoma. Our patient was no different and presented with a polypoidal mass in the left nasal cavity. Apart from the appearance of the tumor, site of the nasal septum involved can be suggestive of schwannoma. The commonest site is the posterior third of the septum with almost 60%, followed by the middle and anterior parts [2]. The patient we are presenting had a tumor originating from the middle part of the nasal septum.

Imaging studies are not helpful in establishing a specific diagnosis of schwannoma [4]. However, it is warranted to define the origin and extent of the disease. This is essential for the planning of the surgery and further treatment. The typical CT findings of nasal septal schwannoma are homogeneous soft tissue density, heterogeneous enhancement with contrast, and bony erosion without destruction. [2,6,8]. In the present case, similar findings were reported except for the sparing of the bone from erosion.

The diagnosis of nasal septal schwannoma is confirmed by the histopathological findings. Schwannomas are composed of spindle cells with two histologically distinct patterns that can be mixed with a demarcation line: Antoni type A with nuclear palisading and Antoni type B with hypocellular areas [9]. In 1951, Ackerman and Taylor ascribed these hypocellular changes to the degeneration or aging of the tumor. Hence, the term “ancient” was coined for schwannoma with decreased cellularity of the connective tissues. However, ancient schwannoma shows similar behavior to other types of schwannoma in terms of growth rate and malignant transformation [10].

In addition, immunohistochemical stains can be useful to corroborate our diagnosis. A neural crest marker antigen, S-100 protein, is shown to have intense and diffuse immunoreactivity by schwannomas [2,5]. Smooth muscle actin and desmin stains were used to exclude neoplasms of myogenic differentiation [11].

In terms of treatment, surgery is the preferred option for nasal septal schwannoma. However, the approaches may vary according to the size and extent of the tumor [1,2,4]. Berlucchi et al. opted for an external approach for their case in the 1990s but advocated for an endoscopic method in their report published in the year 2000. Min et al. in 2017 suggested a transnasal endoscopic approach as the preferred form of surgery. We performed transnasal endoscopic excision of the tumor for our patient and would like to advocate this method for septal schwannomas. There is no conclusive study found to determine malignant transformation, safety margin, and incidence of recurrence [1,2]. Therefore, long-term follow-up is necessary to determine the behavior and natural history of this tumor.

## Conclusions

It is vital to consider nasal septal schwannoma in the differential diagnosis of patients complaining of unilateral nasal obstruction with polypoidal nasal mass, especially the posterior third of the nasal septum. Transnasal endoscopic surgery is the preferred approach for nasal septal schwannoma.

## References

[1] Berlucchi M, Piazza C, Blanzuoli L, Battaglia G, Nicolai P (2000). Schwannoma of the nasal septum: a case report with review of the literature. Eur Arch Otorhinolaryngol.

[2] Min HJ, Hong SC, Kim KS (2017). Nasal septal schwannoma: advances in diagnosis and treatment. J Craniofac Surg.

[3] Ross C, Wright E, Moseley J, Rees R (1988). Massive schwannoma of the nose and paranasal sinuses. South Med J.

[4] Wang LF, Tai CF, Ho KY, Kuo WR, Chai CY (2004). Schwannoma of the nasal septum: a case report.. Kaohsiung J Med Sci.

[5] Min HJ, Kim KS (2017). Differential diagnosis between nasal septal schwannoma and nasal septal neurofibroma. J Craniofac Surg.

[6] Rajagopal S, Kaushik V, Irion K, Herd ME, Bhatnagar RK (2006). Schwannoma of the nasal septum. Br J Radiol.

[7] Pasic TR, Makielski K (1990). Nasal schwannoma. Otolaryngol Head Neck Surg.

[8] Zhou P, Zeng F, Li J, Liu S (2013). Only septal deviation? A tiny schwannoma in the nasal septum. Indian J Otolaryngol Head Neck Surg.

[9] Buob D, Wacrenier A, Chevalier D, Aubert S, Quinchon JF, Gosselin B, Leroy X (2003). Schwannoma of the sinonasal tract: a clinicopathologic and immunohistochemical study of 5 cases. Arch Pathol Lab Med.

[10] Ackerman LV, Taylor FH (1951). Neurogenous tumors within the thorax. A clinicopathological evaluation of forty-eight cases. Cancer.

[11] Molenaar WM, Oosterhuis JW, Oosterhuis AM, Ramaekers FC (1985). Mesenchymal and muscle-specific intermediate filaments (vimentin and desmin) in relation to differentiation in childhood rhabdomyosarcomas. Hum Pathol.

